# Investigating the Flow Dynamics in the Obstructed and Stented Ureter by Means of a Biomimetic Artificial Model

**DOI:** 10.1371/journal.pone.0087433

**Published:** 2014-02-03

**Authors:** Francesco Clavica, Xuefeng Zhao, Motaz ElMahdy, Marcus J. Drake, Xunli Zhang, Dario Carugo

**Affiliations:** 1 Department of Urology, sector FURORE, Erasmus MC, Rotterdam, The Netherlands; 2 Bioengineering Sciences, Faculty of Engineering and the Environment, University of Southampton, Southampton, United Kingdom; 3 Bristol Urological Institute, Southmead Hospital, Bristol, United Kingdom; 4 School of Clinical Science, University of Bristol, Bristol, United Kingdom; 5 Institute for Life Sciences, University of Southampton, Southampton, United Kingdom; 6 Electro-Mechanical Engineering, Faculty of Engineering and the Environment, University of Southampton, Southampton, United Kingdom; 7 Department of Biomedical Engineering, School of Geosciences and Info-Physics, Central South University, Changsha, China; University of Arizona, United States of America

## Abstract

Double-J stenting is the most common clinical method employed to restore the upper urinary tract drainage, in the presence of a ureteric obstruction. After implant, stents provide an immediate pain relief by decreasing the pressure in the renal pelvis (*P*). However, their long-term usage can cause infections and encrustations, due to bacterial colonization and crystal deposition on the stent surface, respectively. The performance of double-J stents - and in general of all ureteric stents - is thought to depend significantly on urine flow field within the stented ureter. However very little fundamental research about the role played by fluid dynamic parameters on stent functionality has been conducted so far. These parameters are often difficult to assess *in-vivo*, requiring the implementation of laborious and expensive experimental protocols. The aim of the present work was therefore to develop an artificial model of the ureter (i.e. ureter model, UM) to mimic the fluid dynamic environment in a stented ureter. The UM was designed to reflect the geometry of pig ureters, and to investigate the values of fluid dynamic viscosity (*μ*), volumetric flow rate (*Q*) and severity of ureteric obstruction (OB%) which may cause critical pressures in the renal pelvis. The distributed obstruction derived by the sole stent insertion was also quantified. In addition, flow visualisation experiments and computational simulations were performed in order to further characterise the flow field in the UM. Unique characteristics of the flow dynamics in the obstructed and stented ureter have been revealed with using the developed UM.

## Introduction

The ureters are conduits conveying urine from kidney into the bladder. They are connected to the renal pelvis through the ureteropelvic junction (UPJ) and to the bladder by means of the vesicoureteric junction (VUJ). In healthy individuals, the propagation of urine is initiated by the pacemaker activity in the renal pelvis [1], [2] which results in ureteric peristalsis (involuntary muscular contractions of the ureteral wall) [3]. However, ureteric obstructions may occur under certain clinical conditions. These include internal occlusions generated by ureteric stones [4], or external compression, for example due to malignant lymph node enlargement [5]. High levels of ureteric obstruction are normally associated with acute pain due to the associated increase in the renal pelvic pressure. Abnormally elevated renal pelvic pressures (*P*>20 cmH_2_O, according to Fung et al. [6]) can potentially lead to irreversible kidney damage. In this clinical situation, ureteric stents are generally inserted into the ureter to restore the drainage of urine [4], [7], [8]. Double-J stent, firstly reported in 1978 by Finney [9], represents the stent architecture most widely adopted in clinical practice. Once in place it extends across the whole ureter length, with side holes positioned at regular intervals through its wall [9]. Stent migration is prevented by the terminal parts of the double-J stent, which curl inside the renal pelvis and bladder. The stent dwell-time can be either short-term, for example in the management of ureteric stones, or long-term in the presence of retroperitoneal fibrosis, inflammatory strictures or pelvic tumours [7]. Despite the extensive clinical experience, complications related to stenting are still frequent, with significant impact on the patient’s quality of life, efficacy of treatment, and cost of patient care [8], [9]. Complications may cause diverse side effects on the treated patient, including discomfort during bladder voiding due to retrograde urine flow along the ureter, abdominal and flank discomfort, and haematuria [9]. Moreover, the placement of a double-J stent is sometimes associated with ureteric dilatation, impaired stone passage [10], [11], thickened ureteric wall [9] and inhibition of peristaltic activity. The latter is thought to be caused by a direct effect of the stent on the pacemaker activity in the renal pelvis and/or on the transmission of peristaltic waves down the ureter [11]–[13]. Persistent irritation [14], stent migration [8], bacterial colonization on the stent surface [15]–[17] and stent encrustation [18] have been identified as the most common causes of stent failure [9]. In this respect, the fluid dynamic field within the stented ureter is thought to play a crucial role in a range of physico-chemical and biological processes including crystals formation and growth, biofilm and bacterial colonization [19], [20]. A few studies have tried to simulate the urine flow in stented ureters, either mathematically [19]–[22] or experimentally [11], [23], [24], but very little fundamental research has been conducted to quantify the effect of fluid dynamic parameters on stent performance [24]. Thus, there is a growing clinical interest in understanding the effect of stent insertion on the urodynamics in the upper urinary tract, and its dependence on clinically relevant factors such as changes in urine viscosity induced by bacterial infections and/or varying severity of ureter lumen occlusion. The main aim of this study is therefore to investigate the flow dynamics in the obstructed and stented ureter using an artificial model which mimics ureter architecture. Using this model, quantitative data on renal pelvic pressure are provided against different physical variables, including (i) urine viscosity, (ii) fluid flow rate and (iii) severity of ureteric obstruction. Results from this study may help understanding how these parameters contribute simultaneously or individually to the progress of stent performance, and thus to the functioning of the upper urinary tract. In this study a double-J stent was used; however the developed model could be further employed to (i) compare the fluid dynamic performance of different stent architectures, (ii) investigate novel stent design options under biomimetic fluidic conditions, (iii) test the effect of stent insertion on the renal pelvic pressure, and implement corrective clinical strategies to prevent kidney damage.

Furthermore, a comprehensive understanding of the flow field in the occluded and stented ureter may help the implementation of corrective strategies to prevent encrustation or bacterial colonization on the stent surface. This applies particularly to regions of the stent, i.e. side holes, which functioning is crucial to maintain urine drainage along the occluded ureter. Notably, it has been suggested that side holes of ureteric stents represent one of the initial anchoring sites for encrusting deposits [25], and inspection of double-J stents retrieved from patients revealed that the majority of the side holes were plugged with crystals and an encircling crystallisation [26]. Therefore, in the present study flow visualisation experiments and computational simulations have been performed in order to investigate the flow dynamics at the interface between stent extra-luminal and intra-luminal compartments, with particular attention devoted to the flow field in close proximity to the side holes of the stent.

## Methodology

### Design and Fabrication of the Ureter Model

Eight ureters were collected from domestic pigs (age: 6–8 months); ethical approval was not required as the specimens were sourced post mortem from local abattoir (with permission of Langford abattoir, University of Bristol, Bristol, UK). Each ureter was detached from the bladder at the VUJ and from the kidney at the UPJ. During the transport and before measurements, the ureters were stored in Krebs solution at 4°C.

**Figure 1:**
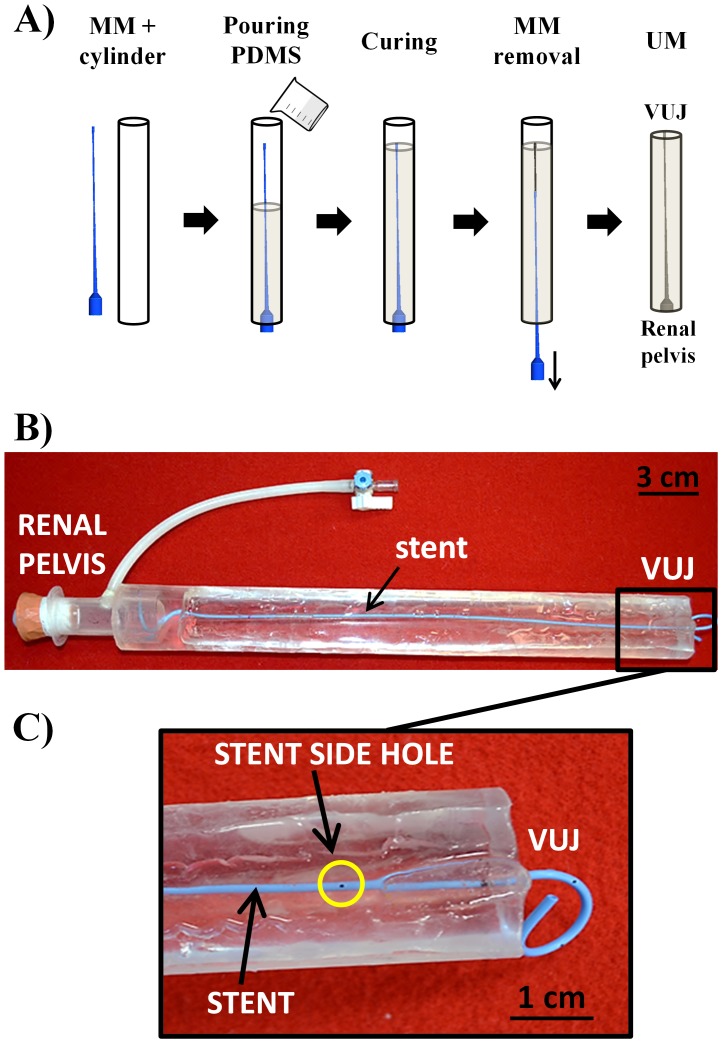
Fabrication of the ureter model. A) Phases of ureter model (UM) fabrication. The male mold (MM) was inserted coaxially into a plastic hallow cylinder. A mixture of PDMS and curing agent was poured into the cylinder and cured at 80°C for 1 hr. The MM was then extracted from the cylinder and the resulting ureter model was further cured at 100°C for 30 min. B) Final UM with double-J stent (blue colour) placed inside. C) Particular of the terminal part of UM, in proximity to the vesico-ureter junction (VUJ). Note the presence of side holes and a curling “J” end of the stent.

Each ureter was cut into 15 equally spaced rings. The inner perimeter (*p_i_* = *πD_i_*) of each ring was measured by opening the segment with a longitudinal dissection; where *D_i_* is the inner diameter of the *i^th^* section. A Computer-Assisted Design (CAD) of pig ureter was performed using ICEM CFD 14.0 (Ansys Inc., USA). The model was based on the experimental values of ureter length (*L*) and *D_i_*, whilst the renal pelvis was modelled with a cylindrical chamber (diameter: 2.0 cm; height: 3.6 cm). From the CAD geometry, a rigid male mold (MM) of the ureter model was produced using a 3D printer (Objet Connex350TM, Stratasys Ltd., USA). The MM was then placed inside and along the axis of a transparent hollow plastic cylinder (diameter: 3.8 cm, length: 33 cm) and a mixture of degassed polydimethylsiloxane (PDMS) precursor and curing agent (10∶1 w/w, Sylgard® 184, Dow Corning Corporation, USA) was slowly poured inside the cylinder (Figure 1A) and cured at 80°C for 1 hr in oven [27]. The MM was then retrieved from the solidified PDMS cylindrical block. Since the PDMS is highly transparent, the final ureter model (UM) was a cylinder with the imprinted ureter geometry, visible from outside. The UM was subsequently placed in the oven for further 30 min at 100°C to achieve complete PDMS curing. The final UM is shown in Figure 1B and 1C.

### Measurements of Renal Pelvic Pressure

**Figure 2:**
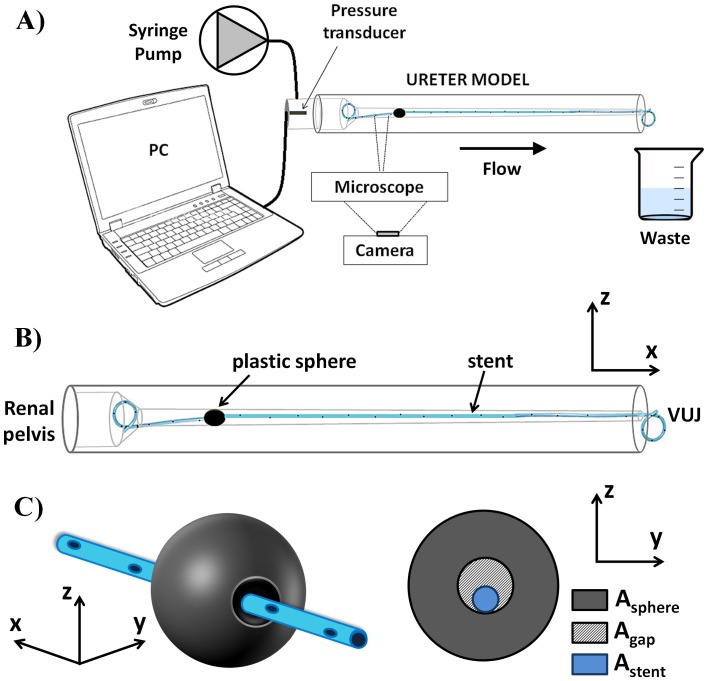
Experimental setup. A) Syringe pump and pressure transducer were connected to the renal pelvis compartment of the ureter model. A microscope and a CCD camera were used for flow visualisation experiments. B) Schematic of the ureter model. The stent was inserted within UM, with its curling ends positioned in the renal pelvis and bladder compartment. C) A plastic sphere was used as a model of ureteric obstruction, with the stent passing through its hole so that the severity of ureteric obstruction (OB%) could be quantified from Eq. 1.

The physiological value of pressure in the renal pelvis is below 20 cmH_2_O [6]. The pressure in the renal pelvis compartment of the UM was measured using a catheter tip pressure transducer (Gaeltec, UK) against three independent variables: volumetric flow rate, fluid dynamic viscosity and severity of ureteric obstruction. Pressure recordings were performed using an in-house developed software, written in LabVIEW environment (National Instrument, USA). The bladder compartment was the output of the UM, and it was modelled as an open end (P_bladder_ = 0 cmH_2_O). The experimental setup is shown in Figure 2. A 41 cm long double-J stent (Contour™, Boston Scientific/Microvasive, USA) was inserted in the UM, following the recommended clinical procedure for insertion. The stent was placed in such a way to have its curling ends within the renal pelvis and the bladder compartment, respectively. The inner diameter of the stent was 1.28 mm and the outer diameter 2.08 mm. In order to investigate the effect of urine viscosity changes on renal pelvic pressure (e.g. occurring during urine infections or kidney malfunctioning), the urine in the UM was modelled with a solution of deionized water (Milli-Q, EMD Millipore Corporation, USA) and glycerol (Sigma Aldrich Co., UK) at different concentrations. Six solutions were produced, each having a different dynamic viscosity corresponding to the following mass proportion (%) of glycerol in distilled water: 0, 10, 20, 30, 40 and 50 (coherently with Segur et al. [28]). Viscosity values are reported in Table 1.

**Table 1:** Summary of the experimental parameters values investigated in the present study to test the effects on the renal pelvic pressure.

**Flow rate (ml/min)**	5		10		15		20	
**Fluid Viscosity (cP)**	1	1.3		1.7	2.5		3.7	6
**OB%** a	81	84	88	91	94	96	99	100

a Severity of obstruction (calculated using Eq. 1).

A syringe pump (KD Scientific, UK) was connected to the renal pelvis compartment (Figure 2) to simulate urine production from the kidney. Four different flow rates (*Q*) were enforced (Table 1), within the physiological range for pigs (0 – 20 ml/min, according to Tofft et al. [29]).

To investigate the effect of an occlusion in the upper part of UM on the renal pelvic pressure, obstructions were modelled using eight identical plastic spheres. The severity of obstruction was regulated by drilling circular holes along one axis for each sphere separately, allowing for the stent to pass through the sphere hole (Figure 2C). Despite it does not simulate faithfully the physiological situation, the approach adopted here allows to generate a controlled and measurable occlusion of the UM lumen. One plastic sphere each time was used for a given severity of obstruction. To generate the obstruction, the sphere was pushed into the UM lumen until no further translation was allowed. In this way, an obstruction was generated at a given *i^th^* longitudinal coordinate, positioned between the 5^th^ and the 6^th^ side hole of the stent (counting from the renal pelvis).

Thus, it could be assumed that *A_sphere_* = *A_i_*, where *A_sphere_* = π·*D_sphere_* ^2^/4 is the cross-sectional area of the plastic sphere (*D_sphere_* is the sphere diameter, and is equal to 6.07±0.04 mm) and *A_i_* = π·*D_i_* ^2^/4 is the local cross-sectional area of UM lumen (at the *i^th^* axial coordinate). The percentage severity of the generated ureteric obstruction (OB%) was calculated as follow:
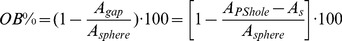
(1)


Where *A_s_* = 3.4 mm^2^ is the stent cross-sectional area, *A_PShole_* is the cross-sectional area of the hole in the sphere, and represents the only variable with which to control the severity of ureteric obstruction. *A_gap_* is the space between the hole of the plastic sphere and the external surface of the stent (Figure 2C). As an example, OB% = 100 corresponds to *A_gap_* = 0, with the all fluid passing through the stent lumen in correspondence to complete obstruction (absence of extra-luminal flow at the obstruction).

A linear regression based on the least squares method was fitted to *P vs Q*, and *P vs* OB% experimental data.

### Flow Visualisation Experiments

The UM was placed on the stage of a fluorescent microscope (IX71, Olympus Corporation, Japan). Microscope focus was set in correspondence to the midplane of the UM (i.e., in the *z*-direction, Figure 2B). Small adjustments of the focal plane were performed in some cases, in order to allow for a clear and comprehensive visualisation of the flow field in close proximity to the stent side hole and in the extra-luminal space of the stent. The focal distance could also be regulated by cutting off part of the PDMS to improve the visual accessibility to the UM lumen. Fluorescent polystyrene beads (15 *µ*m diameter; Invitrogen, USA) were added to the working fluid. Beads were exposed to fluorescent light (excitation wavelength, *λ_ex_* = 441 nm) and emitted light with a wavelength, *λ_em_*, of 486 nm. By using an optical filter, the fluorescent images of beads were acquired by a CCD camera (NIKON 5100, Japan) with a spatial resolution of 1392×1024 pixels×pixels and inter-frame time interval of 20 ms. A 4x magnification objective was employed for this purpose. Images were acquired in close proximity to a side hole of the stent, positioned just before/after the plastic sphere.

### Computational Fluid Dynamic (CFD) Simulations

With the aim of investigating further the urine flow dynamics in the obstructed and stented ureter, a simplified two-dimensional (2D) numerical model was developed.

As for experimental flow visualisation studies, we focused our attention on a region of the fluidic domain located in close proximity to a side hole of the stent, positioned after the obstruction (6^th^ hole, counting from the renal pelvis curled J). ICEM CFD 14.0 (Ansys Inc., USA) was employed for construction and meshing of the two-dimensional model geometry. In particular, the geometry reproduced a segment of the extra-luminal region of the stent positioned in proximity to a stent side hole, and located after the obstruction.

**Figure 3:**
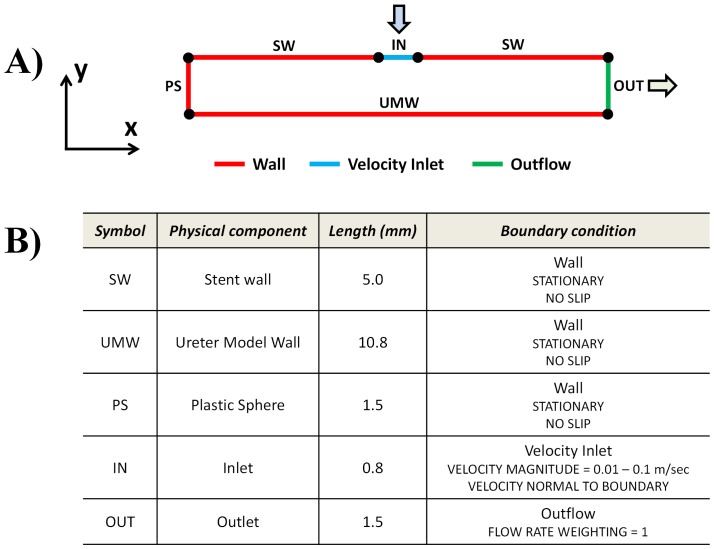
Schematic of the computational domain and boundary conditions. A) Schematic representation of the two-dimensional computational domain. Colours correspond to different boundary conditions: red wall boundary; green outflow; light blue velocity inlet. B) Summary of the geometrical characteristics of the computational domain, and boundary conditions applied to each individual edge.

Figure 3 shows a schematic of the computational domain and related boundary conditions. The stent side hole was modelled with an edge, having the same length of the experimentally-assessed hole diameter (0.8 mm). The distance between the stent outer wall and the UM inner wall was set to 1.5 mm, which corresponds to the distance taken at the midplane, assuming that stent and UM are coaxial within the length segment investigated. The distance between ureter occlusion and the stent side hole was instead set to 5 mm, conforming to the experimental conditions.

The geometry was meshed using quadrilateral elements, with mesh element size of 0.01 mm (corresponding to a total of 496′250 elements). The following mass conservation (Eq. 2) and momentum conservation (Eq. 3) equations were solved over the computational flow domain, using Ansys Fluent 14.0 (Ansys Inc., USA):
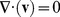
(2)

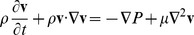
(3)where *v*, *ρ*, *μ* and *P* represent fluid velocity, density, dynamic viscosity and pressure, respectively. The working fluid was assumed to be incompressible and Newtonian, with a density of 1000 kg/m^3^ and a dynamic viscosity of 0.001 Pa⋅sec. The flow was assumed to be steady and laminar. The Semi-Implicit Method for Pressure-Linked Equations (SIMPLE) algorithm was employed for solving the governing equations. Stent/ureter walls were assumed to be rigid with a no-slip flow boundary condition imposed on each wall (SW and UMW, Figure 3A and 3B). A velocity boundary condition was applied at the stent side hole (IN, Figure 3A and 3B). Velocity value was determined from experiments using fluorescent tracers, in which microscope focus was set in correspondence to the midplane (i.e., in *z*-direction) of the stent side hole. An outflow boundary condition was imposed at the distal side of the fluidic domain (OUT, Figure 3A and 3B), while a wall boundary with no-slip flow condition was imposed in correspondence to the plastic sphere (conforming to a complete occlusion of the ureter lumen, i.e. OB% = 100) (PS, Figure 3A and 3B).

## Results

### Ureter Geometry

**Figure 4:**
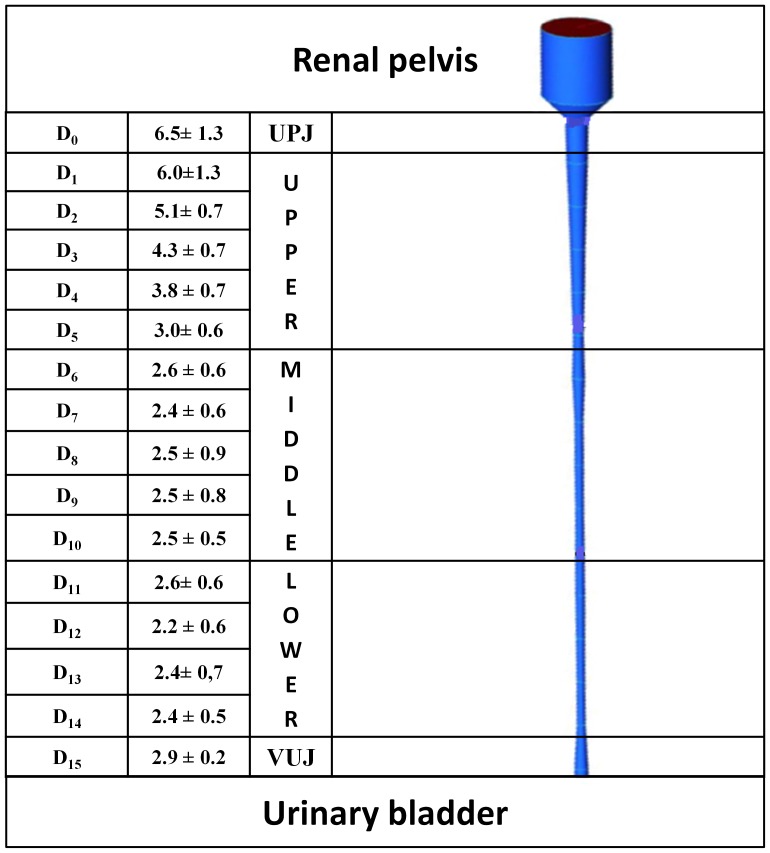
Reconstruction of pig ureter geometry. Average pig ureter internal diameters (*D_i_*, in mm; N = 8), along the longitudinal coordinate *i* (*i* = 0 at UP; *i* = 15 at VUJ) classified in Upper, Middle and Lower ureter (left). UPJ = ureteropelvic junction, VUJ = vesicoureteric junction. On the right, computer-aided design of the ureter employed as an input geometry for 3D printing.

Average values of measured diameters along the longitudinal axis of the pig ureter are shown in Figure 4. The average length of the ureters was *L* = 289±20 mm. Since *D_sphere_* = 6.07±0.04 mm, we estimated that the sphere created an upper obstruction in UM at *D_0–1_* (Figure 4).

### Renal Pelvic Pressure *vs* Urine Properties and Severity of Obstruction

**Figure 5:**
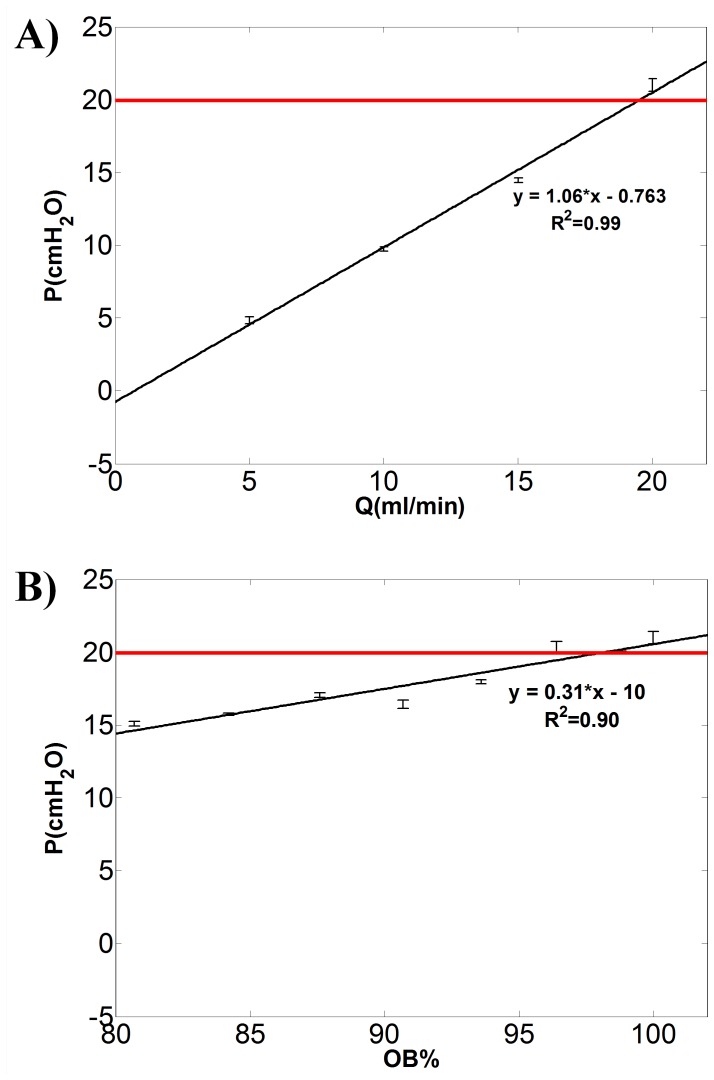
Renal pelvic pressure *vs* urine properties and severity of obstruction. Renal pelvic pressure (*P*) in the ureteric model increases linearly with A) flow rate (*Q* in ml/min) and B) severity of obstruction in the upper UM (OB%, calculated using Eq. 1). Fixed values of OB% = 100 and *Q* = 20 ml/min were considered for the experimental data reported in A) and B), respectively. The fluid dynamic viscosity is equal to 1 cP. The equation of the least square regression line, the R-squared values and error bars are reported (N = 3). The slope of regression line in panel A represents the hydraulic resistance of the system. The horizontal red line indicates the critical value of renal pelvic pressure (*P* = 20 cmH_2_O).

Representative results illustrating the dependence of UM renal pelvic pressure on *Q* and OB% are shown in Figure 5. Figure 5A refers to a fixed OB% = 100 in upper UM and *μ* = 1 cP (i.e. distilled water was employed as working fluid).

The slope of the regression line in Figure 5A represents the hydraulic resistance of the system (*m* = 1.06 cmH_2_O/(ml/min)). It can be observed that the renal pelvic pressure exceeds the critical value of 20 cmH_2_O [6] only at one experimental condition, corresponding to *Q* = 20 ml/min. Renal pelvic pressure *vs* severity of obstruction (OB%) is reported in Figure 5B, at a fixed *μ* = 1 cP and *Q* = 20 ml/min. It can be observed in this case that renal pelvic pressure exceeds the critical value at three experimental conditions, corresponding to OB% = 96, 99 and 100.

The values of hydraulic resistance (*m*) for the full range of combinations of *μ* and OB% can be derived from the slope of the *P*–*Q* interpolating functions, as reported in Figure 5A. Values of *m* are reported in Table 2, and are observed to increase with increasing OB% from 0 to 100 (i.e. from top to bottom) and with increasing *μ* from 1 to 6 cP (i.e. from left to right). The large majority of R-squared values are close to 0.9, showing an evident linear relationship between *P* and OB%, and between *P* and *μ*. The lower values of *m* and R-squared are associated to the unobstructed configuration (OB% = 0, absence of plastic sphere and stent in UM), in which increments of *μ* have less effect on *P*.

**Table 2:** Hydraulic resistances (in cmH_2_O*60 s/ml) measured from the slope (*m*) of the linear regression (R-squared values, R^2^, are also reported) of renal pelvic pressure *versus* flow rate values (example in Figure 5A) for each combination of viscosity and severity of obstruction (OB%).

	Viscosity (cP)											
OB%	1		1.3		1.7		2.5		3.7		6	
	*m*	R^2^	*m*	R^2^	*m*	R^2^	*m*	R^2^	*m*	R^2^	*m*	R^2^
0	0.046	0.93	−0.004	0.51	0.007	0.60	0.028	0.95	0.03	0.269	0.323	0.689
only stent	0.675	0.99	0.642	0.999	1.102	0.998	1.210	0.99	1.481	0.999	2.909	0.999
84	0.779	0.99	0.957	1	0.805	0.887	1.682	0.99	2.443	0.999	3.956	0.99
88	0.899	0.99	0.989	0.998	1.246	0.999	1.645	1.00	2.052	0.999	3.533	0.999
91	0.781	0.98	0.891	0.971	1.266	1.266	1.649	0.99	2.091	0.999	3.419	1
94	0.841	0.99	1.206	0.998	1.295	0.999	1.912	0.99	2.868	0.99	4.038	0.99
96	1.046	0.98	1.303	0.999	1.447	0.998	2.152	0.99	2.84	0.999	4.591	0.99
100	1.06	0.99	1.329	0.704	1.603	0.998	2.065	0.99	3.115	0.999	5.078	0.99

**Figure 6:**
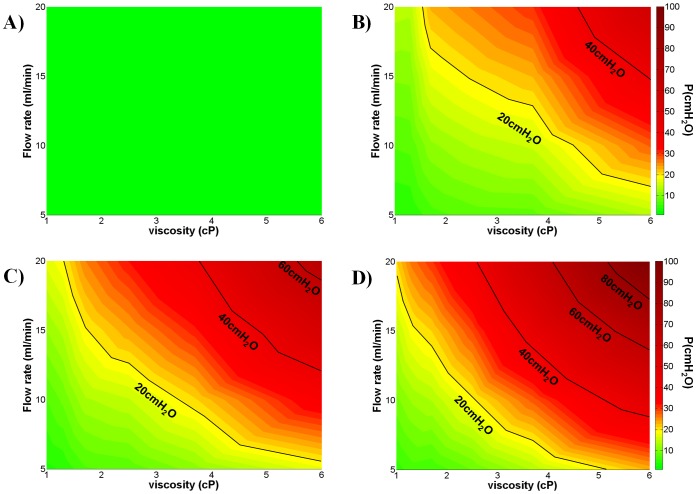
Dependence of UM renal pelvic pressure on fluid flow rate, dynamic viscosity and severity of obstruction. Colourmaps show the dependence of ureteric model (UM) renal pelvic pressure on both the fluid flow rate (*Q* in ml/min, y-axis) and the fluid dynamic viscosity (*μ* in cP, x-axis). Colours correspond to different pressure values (in cmH_2_O) reported in the colourbar on the right hand side. A) OB% = 0, corresponding to unobstructed UM (absence of both plastic sphere and stent). B) refers to the condition of obstruction-causing effects, due to the stent only (absence of plastic sphere). Adding the plastic sphere, two values of severity of obstruction were considered: C) OB% = 88 and D) OB% = 100 (stent+plastic sphere, with hole size according to Eq. 1). Iso-pressure lines (in cmH_2_O) are reported (black lines). The green area corresponds to the safe region (*P*<15 cmH_2_O), the yellow area to the warning region (15<*P*<20 cmH_2_O) while the orange/red area to the dangerous region (*P*>20 cmH_2_O) for correct kidney functioning. N = 3.

Colourmaps of Figure 6 show the hydraulic pressure in the UM renal pelvis as a function of the fluid dynamic viscosity (x-axis, in cP) and volumetric flow rate (y-axis, in ml/min). Values have been derived from linear interpolation of the experimental data points. The colours are representative of the pressure values. The green area corresponds to the ‘safe region’ (*P*<15 cmH_2_O), the yellow area to the ‘warning region’ (15 cmH_2_O <*P*<20 cmH_2_O), and the orange/red area to the ‘dangerous region’ (*P*>20 cmH_2_O, according to Fung et al. [6]) for the kidneys. Figure 6A refers to the condition of unobstructed ureter; notably the kidney pressure is below the critical value even at the highest values of fluid viscosity and flow rate. In this situation, the minimum pressure (at *Q* = 5 ml/min and *μ* = 1 cP) is 0.4±0.08 cmH_2_O, while the maximum pressure (at *Q* = 20 ml/min and *μ* = 6 cP) is 1.4±0.11 cmH_2_O. Figure 6B refers to the condition of stented ureter, in the absence of plastic sphere positioned in the upper UM lumen. The insertion of the sole stent causes a significant increase in hydraulic resistance, and both warning and dangerous regions appear in the colourmap at the higher values of *Q* and *μ*. Wider and more progressive (occurring at lower *Q* and *μ*) warning and dangerous regions were found, in the presence of plastic sphere, at OB% = 88 (Figure 6C) and OB% = 100 (Figure 6D).

### Experimental and Computational Characterisation of the Fluid Dynamic Field

Flow visualisation experiments were carried out to characterise the fluid dynamic environment in the UM, under a range of simulated clinically-relevant conditions including volumetric fluid flow rate and severity of UM obstruction. In particular, our attention focused on the fluid dynamic field in close proximity to a side hole of the stent, located either before or after UM occlusion. Video S1 shows the flow behaviour of 15 *µ*m fluorescent tracers in a region positioned just prior to the occlusion (5^th^ stent side hole), at *Q* = 1 ml/min and OB% = 96; Video S2 instead refers to a region positioned just after the occlusion (6^th^ stent hole). In Video S3 an example of flow behaviour in a region at a distance of 15 cm from the occlusion (close to VUJ) is also provided (OB% = 96, Q = 5 ml/min).

**Figure 7:**
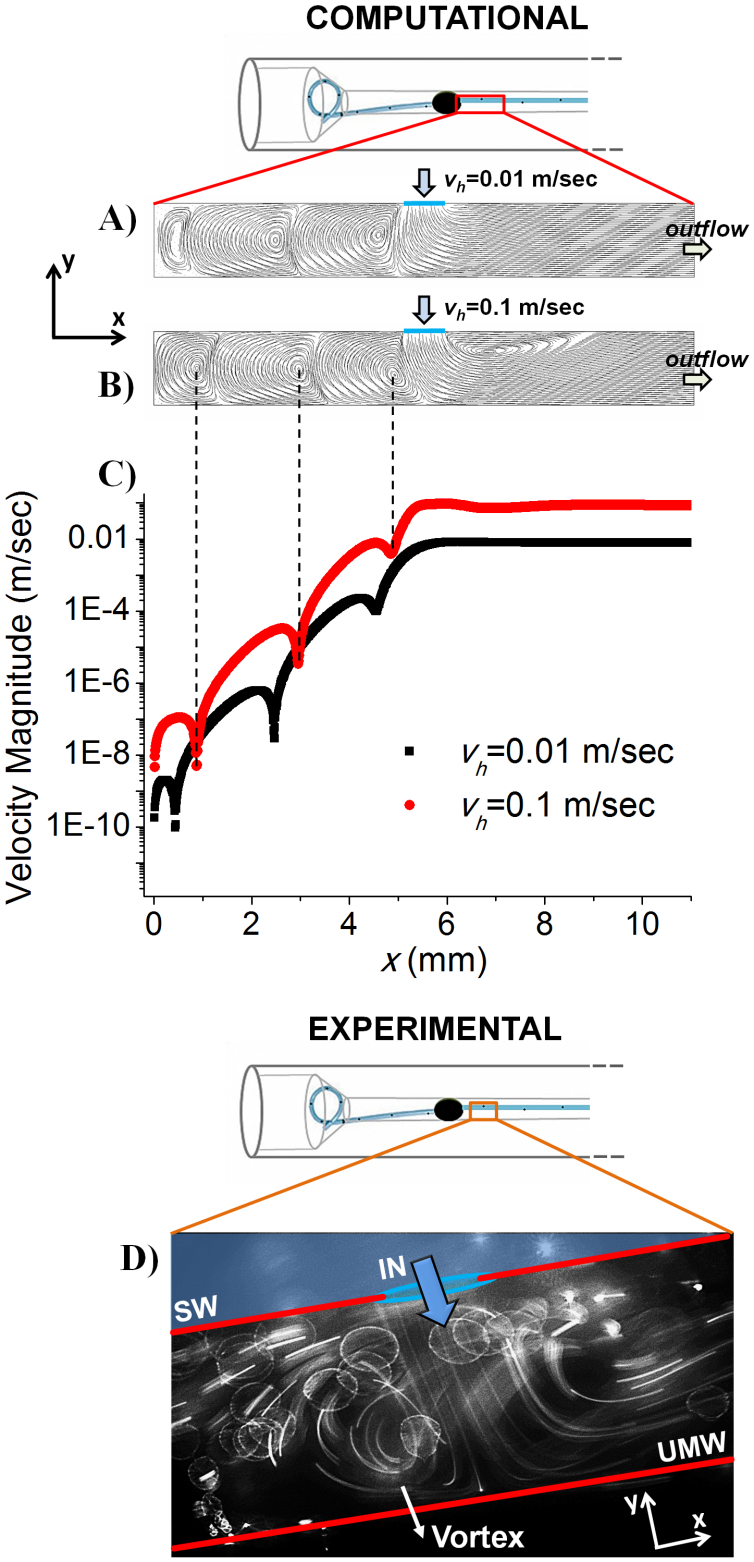
Two-dimensional numerical model of the flow field in close proximity to a stent side hole. A–B) Numerical fluid pathlines in a region of the extra-luminal space of the stent positioned in close proximity to the 6^th^ side hole, located after the plastic sphere. The mean velocity of the fluid exiting the stent side hole was set to: A) 0.01 m/sec and B) 0.1 m/sec. C) Magnitude of fluid velocity (in Log scale) along the centreline of the fluidic domain (in the *x*-direction), at *v_h_* = 0.01 m/sec (black squares) and 0.1 m/sec (red circles). *x* = 0 mm corresponds to the position of UM obstruction (i.e. plastic sphere) and *x* = 10.8 mm to the outflow boundary. Velocity minima correspond to the approximate position of eddies centre, as indicated by the black dashed lines. Changes in eddies velocity and size with increasing *v_h_* can be appreciated. D) formation of laminar eddies has been observed experimentally using fluorescent tracers, thus qualitatively corroborating with the numerical results. The direction of fluid flowing from the intra-luminal to the extra-luminal region of the stent is indicated by the blue arrow. SW = Stent wall; UMW = Ureter model wall.

The flow pattern in proximity to the 6^th^ stent side hole (located after the occlusion) has been further investigated by means of a simplified 2D numerical model. In this respect, Figure 7A and 7B show fluid pathlines in the extra-luminal region of the stent determined computationally, at two different mean velocities of the fluid exiting the stent side hole (*v_h_*), corresponding to *v_h_* = 0.01 m/sec (Figure 7A) and *v_h_* = 0.1 m/sec (Figure 7B), respectively. Velocities have been determined experimentally by manually tracking beads motion over subsequent image frames. Qualitative agreement between experiments and numerical simulations can be appreciated (Figure 7D). Figure 7C shows the fluid velocity magnitude along the centreline of the fluidic domain (in the *x*-direction) at *v_h_* = 0.01 m/sec and 0.1 m/sec, as determined computationally.

## Discussion

Urine drainage through a stented ureter is a multifactorial and complex process depending on renal pelvic pressure, bladder pressure and severity of ureteric obstruction, stent internal/external diameter and length, size and number of stent holes and urine physical properties (i.e. viscosity) [9]. In a stented ureter, urine can flow either in the extra-luminal space (located between the outer stent wall and the inner wall of the ureter) or in the intra-luminal space of the stent. Only few studies have attempted to model dynamically ureteric stents exposed to flow *in vitro* [30], [31]. It has been demonstrated that increasing stent inner diameter results in increased intra-luminal flow [24]. Furthermore, the presence of side holes through the stent wall has been associated with a 40–50% increase of urine drainage compared to a hole-free stent [9], [10]. Presence of side holes has also been associated with a reduction of the vesicorenal reflux up the stent, since most of the backflow from the bladder is dissipated through the holes before reaching the renal pelvis [23], [32], [33]. However, whilst several studies have tried to describe the fluid dynamics in the stented ureter qualitatively, to the best of our knowledge quantitative data are still missing. A quantitative understanding of the causes leading to kidney damage, urine infection and/or stent encrustation represents an essential subject of investigation. In this respect, the developed biomimetic and transparent model represents a first attempt to simulate closely the fluid dynamic environment in the obstructed and stented ureter. From Figure 5 and Table 2 we have demonstrated that the relation between renal pelvic pressure *vs* urine viscosity, flow rate and severity of obstruction is linear in the large majority of cases examined. The minimum hydraulic resistance (0.007 cmH_2_O/(ml/min)) was measured in the unobstructed UM, and it was shown not to change significantly with varying urine viscosity.

Values of OB% reported in Table 1 refer only to the severity of obstruction at *D_0–1_*, where a controlled occlusion of UM lumen was generated. However, the stent alone (*D_s_* = 2.08 mm) introduces a relevant obstruction distributed along the whole ureter length, which is particularly significant in the section of UM with the lowest diameter (*D_12_* = 2.2 mm, in Figure 4). The severity of this distributed obstruction (due solely to the stent), at a generic *i^th^* position (OB_i_%), can be calculated by assuming *A_PShole_* = *A_sphere_* = *A_i_* in Eq. 1, as follows:
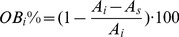
(4)


**Figure 8:**
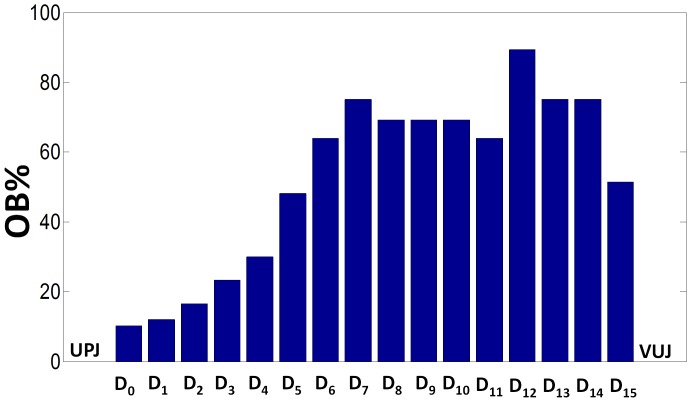
Severity of UM obstruction induced solely by stent insertion. Severity of obstruction (OB_i_%) calculated along the longitudinal coordinate of the UM (*D_0–15_*, see Figure 4 for reference) introduced by the double-J stent only (absence of plastic sphere). For the calculations, Eq. 4 was considered. UPJ and VUJ indicate the urteropelvic and vesicureteric junctions, respectively.

Figure 8 shows, in the form of histograms, the distribution of OB_i_% along the ureter length, solely due to the presence of the stent in UM. OB_i_% reaches its maximum (approximately 90) at *i* = 12.

The fact that the stent alone introduces a relevant obstruction appears also evident by looking at the increased values of resistance in Table 2 passing from the unobstructed situation (OB% = 0) to the stent insertion (‘only stent’), and by the appearance of the warning and dangerous regions in Figure 6B. The above observations may suggest that deployment of a ureteric stent should be considered with care, since the distributed obstructions introduced by the stent itself should be counterbalanced by the advantage of providing a stable path for the urine drainage.

The UM may be of help to clinicians to understand and quantify the individual and combined effect of the modelled physical variables on the renal pelvic pressure, under a range of clinically relevant conditions. Figure 6, as an example, provides an intuitive identification of the combined values of urine flow rate and viscosity for which the renal pelvic pressure is located below or above the iso-pressure line at 20 cmH_2_O, which correspond to normal kidney function (“safe area”) or potential kidney damage, respectively. Moreover, Table 2 clearly illustrates how a small increase of urine viscosity (values from left to right) and severity of obstruction in the upper UM (values from top to bottom) strongly affect the hydraulic resistance of the system, with significant effect on the renal pelvic pressure. The comparison between Figure 6C and Figure 6D, for example, clearly illustrates how a small increase in the severity of obstruction (e.g. from 88% to 100%) significantly affects the size of the “safe area” with a more limited range of variations, for both *μ* and *Q*, in the case of higher obstruction values.

In addition to the possibility of studying the hydraulic behaviour of a stented ureter as a whole system, the developed UM allowed for the investigation of the local fluid dynamic field at regions of interest within the model. This is due to the optical transparency of the UM, and the possibility of coupling it with microscopy-based equipment. Notably, understanding the local dynamics of urine flow in a stented ureter is of crucial importance to identify the potential physical factors leading to stent encrustation or biofilm formation and growth on the stent surface. In this respect, it has been observed that in the presence of a severe obstruction of the ureter lumen the insertion of a double-J stent allows for effective drainage of fluid through the ureter and by-passing of the obstruction. This is manifested by the number of fluorescent beads flowing into the stent lumen *via* the side hole of the stent located prior to the obstruction (Video S1). The presence of residual fluid flowing in the extra-luminal space of the stent is also detectable, due to the incomplete nature of the occlusion (Video S1). Furthermore Video S3 has shown that a number of fluorescent beads, flowing inside the stent lumen, can also be detected in regions distant from the occlusion (i.e. 15 cm after the obstruction).

Interestingly, the onset of peculiar fluid dynamic patterns has been detected in the extra-luminal space of the stent located just after the occlusion (Video S2), which have been further characterised by means of a simplified two-dimensional numerical model. Numerical results show the formation of laminar counter-rotating eddies in the region of the extra-luminal space located between the occlusion and the stent side hole. In particular, at complete ureter lumen occlusion (i.e., OB% = 100), numerical results predict that eddies size and velocity depend on the mean velocity of the fluid exiting the side hole of the stent (*v_h_*). With increasing *v_h_* both size/position (Figure 7A and 7B) and velocity (Figure 7C) of the formed eddies was observed to vary. Figure 7C shows the magnitude of fluid velocity along the centreline of the 2D domain (i.e., in the *x*-direction), at *v_h_* = 0.01 m/sec and 0.1 m/sec. Velocity minima correspond to the position of eddies centre. Results show that increasing the velocity of the fluid exiting the side hole of the stent from 0.01 m/sec to 0.1 m/sec results in increased eddies velocity. Numerical results thus suggest that the strength of eddies formed in the extra-luminal space of the stent increases with increasing the volumetric flow rate (at a given severity of obstruction) or with increasing the severity of obstruction (at a given flow rate).

The size and position of the formed eddies was also observed to depend on *v_h_*. In particular, increasing *v_h_* caused a translation of eddies centre towards the stent side hole, which was accompanied by increased size of the eddy located nearby the ureteric occlusion. Changes in eddies position and size may have important implications on the spatial location and extent of biofilm/crystals adhesion on the stent surface.

Importantly, experimental flow visualisation qualitatively corroborated with the numerical results, confirming the formation of eddies in the extra-luminal space of the stent (Figure 7D).

To the best of our knowledge, this is the first study to have investigated the fluid flow fields in an occluded and stented ureter model *in situ*. The observation of laminar eddies in the extra-luminal space of the stent is unique and merits further investigations, particularly on the role that these fluid flow patterns may play in the formation and growth of crystals or bacterial biofilms. Similarly to plaque formation in carotid artery, as suggested by Liepsch et al. [34], also in stented ureters the formation of eddy structures may cause particles (crystals and/or bacteria) being trapped for a certain time before they are washed away. This seems to be a reasonable explanation for the formation of particle aggregations which then trigger bacterial adhesion and/or encrustation processes nearby the side holes of the stent [25], [26]; thus opening new exciting perspectives in the field of stent architecture design and optimization. We anticipate that this will represent the subject of future investigations.

### Limitations

Ureter distensibility has not been taken into account in this work as, due to the large number of physical variables involved, we initially designed a rigid UM. However, the modelling of ureter distensibility is non-trivial, since regional differences exist in elastic wall properties [35]. Compared to a rigid conduit, in a distensible ureter we would expect increased inner diameter particularly of the upper tract (which is more compliant [35]). However, this may not cause a significant change in the total pressure drop, compared to the rigid case, as most of the hydraulic resistance can be attributed to the ureter lumen occlusion, and to the middle and distal tracts (which are less compliant). Furthermore, increased accumulation of extracellular collagen has been reported in dilated ureters, causing increased wall stiffness and reduced distensibility [36], [37]. In order to quantitatively assess the effect of ureter distensibility, future studies will focus on the development of distensible UM, which could be achieved by precisely dosing PDMS precursor and curing agent to match the physiologic/pathologic values of ureter distensibility.

Peristaltic activity of ureteric walls has also been neglected in our modelling. Since the stenting normally results in a pronounced reduction of ureteric peristalsis (particularly in the long-term) [7], [19], we assumed that the modelled conditions (i.e. stationary walls) can still resemble the fluid dynamic environment of a stented ureter. Furthermore the bladder has been modelled as open end; in future investigations the control of the pressure inside the bladder compartment may improve the understanding of the reflux of urine from bladder to kidneys, which is also a common problem of stented ureters.

### Summary and Key Conclusions

The present work has provided a technologic platform aimed at understanding the urine flow dynamics within obstructed and stented ureters as an alternative to laborious and expensive *in-vivo* experimental protocols. The preliminary data obtained using the developed UM have covered different simplified conditions (i.e. unobstructed/obstructed and stented ureter, changes of urine viscosity and flow rate) and can be extended to simulate more complex physiologic/pathologic conditions. Clinicians may benefit from the developed platform to understand and quantify the individual and combined effects of the modelled variables on the urine drainage and kidney functioning (i.e. renal pelvic pressure) in a stented ureter. The UM has also provided interesting information on the local fluid dynamics (i.e. formation of eddies in the extra-luminal space). Future developments will aim to identify the relation between local fluid dynamic phenomena and biofilm formation or crystals deposition on the stent surface.

## Supporting Information

Video S1 **Flow visualisation before the obstruction.** Flow visualisation shows the fluid entering the stent side hole before the obstruction.
(WMV)

Video S2 **Flow visualisation after the obstruction.** Flow visualisation shows the fluid exiting the stent side hole after the obstruction, with formation of laminar eddies.
(WMV)

Video S3 **Flow visualisation at a distance of 15 cm after the obstruction.** Flow visualisation shows the fluid entering the stent side hole at a distance of 15 cm after the obstruction.
(WMV)
